# Analysis of the Weekend Effect on Mortality, Diagnostic Coronary Angiography, and Percutaneous Coronary Intervention in Acute Myocardial Infarction Across Rural US Hospitals

**DOI:** 10.7759/cureus.53751

**Published:** 2024-02-07

**Authors:** Mirza Faris Ali Baig

**Affiliations:** 1 Internal Medicine, Asante Three Rivers Medical Center, Grants Pass, USA

**Keywords:** mortality, early pci, early diagnostic angiogram, percutaneous coronary intervention, diagnostic coronary angiography, rural hospital, weekend effect

## Abstract

Background: Rural hospitals face several unique challenges in delivering healthcare to an underserved population. Achieving time-sensitive goals in a resource-scarce facility is often a difficult task without the right team at hand. Resources are further depleted on the weekends, exposing understaffed hospitals to poorer outcomes. Acute myocardial infarction (AMI) mortality depends on timely diagnosis and intervention. It is unknown to what extent resource shortages impact rural hospitals during weekends and how they affect AMI mortality.

Methods: This cross-sectional study was performed on patients admitted on weekends with AMI using the National Inpatient Sample (NIS) 2019. Patients with type II non-ST-elevation myocardial infarction (NSTEMI) and missing information were excluded. The rates and timing of in-hospital diagnostic coronary angiograms, PCIs (percutaneous coronary interventions), and in-hospital mortality were studied. Regression models were used for data analyses.

Results: A total of 161,625 patients met the inclusion criteria (58,690 females (36%), 114,830 Caucasians (71%), 17,910 African American (11%), 13,920 Hispanic (8.6%); mean (SD) age, 66.5 (0.5) years), including 47,665 (29.5%) ST-elevation myocardial infarction (STEMI) and 113,960 (70.5%) NSTEMI. Patients admitted to rural hospitals were less likely to undergo diagnostic coronary angiogram (adjusted odds ratio (aOR), 0.69; CI, 0.57-0.83; *p*<0.001) and PCI (aOR, 0.83; CI, 0.72-0.96; *p* 0.012). Rural hospitals had lesser odds of early diagnostic angiograms (aOR, 0.79; CI, 0.67-0.95; *p*<0.05) and PCI (aOR, 0.78; CI, 0.66-0.92; *p*<0.05) within 24 hours. The mortality difference between rural and urban hospitals was not significant (aOR, 1.08; CI, 0.85-1.4; *p* 0.52).

Conclusions: Diagnostic coronary angiograms and PCI are performed at a lesser rate in rural hospitals during weekends. This trend did not affect rural AMI mortality.

## Introduction

Coronary artery disease (CAD) is the leading cause of death in the United States and globally [1]. The mortality associated with CAD has declined over the last few decades due to technological advancement and well-defined time-sensitive protocols [2]. Acute coronary syndrome outcomes are better with early reperfusion therapies and door-to-balloon (D2B) time of less than 90 minutes [3]. Percutaneous coronary intervention (PCI)-capable hospitals are monitored on their D2B times [4]. Therefore, efforts are made at the first medical contact to expedite transfer to a capable facility for early initiation of reperfusion therapy and prevent further ischemic injury.

Despite implementing strict policies to decrease CAD mortality, several barriers exist to achieve the defined goal in rural hospitals. While most of the United States is rural, it is estimated that 19.3% of the total population lives there and only one-tenth of providers practice in these areas [5]. Rural populations are more likely to be older, uninsured, and with limited medical literacy. These patient factors and limited access to primary and specialty care culminate in poor health outcomes [6]. The disparities in health outcomes have further widened in the last three decades, leading to the issuance of a call for action by the American Heart Association and American Stroke Association in 2020 [7].

The phenomenon known as the weekend effect is widely discussed and studied both in the United States and globally, and it is linked with higher mortality rates [8]. While some studies attribute the increased mortality to lower resource availability [9], other researchers advocate for the idea that the mortality difference stems from a selection bias, where only the sickest patients are admitted during the weekends [8]. Healthcare facilities typically operate with reduced staff on weekends, resulting in limited availability of qualified personnel, and strict triaging policies that prioritize life-threatening conditions until full service is restored [10]. Some studies reported adverse prognostic outcomes over the weekend, while others indicated no significant impact on mortality [11,12]. For time-sensitive disease processes such as acute myocardial infarction (AMI) or cerebrovascular attack, the accessibility of such resources plays a crucial role and directly impacts morbidity and mortality [13,14].

Rural healthcare facilities are more vulnerable to understaffing, especially during weekends, potentially exacerbating overall mortality rates. Despite extensive discussions about outcome disparities based on urban-rural settings and the weekend effect, no study has specifically examined the impact of the weekend effect on rural mortality. This study is designed to analyze the weekend effect on the utilization of diagnostic coronary angiography and PCI, mean time taken to perform these procedures, in-hospital mortality, and resource utilization in AMI patients treated in rural hospitals across the United States.

## Materials and methods

This study represents a cross-sectional observational analysis involving adult patients hospitalized with the diagnosis of AMI in the United States. The study utilized data extracted from the National Inpatient Sample (NIS) from January 1 to December 31, 2019. The NIS is a part of the Healthcare Cost and Utilization Project (HCUP), overseen by the Agency for Healthcare Research and Quality (AHRQ) [15]. The NIS sampling frame collected data from 49 state organizations, estimating the coverage of 97% of discharges from non-federal US hospitals, encompassing 98% of the US population. The NIS includes a stratified sample of 20% of discharges from all HCUP-participating hospitals, totaling seven million discharge records, which approximates to 35 million discharges in 2019 when discharge weights are applied.

Study patients

Adult patients (≥18) admitted non-electively over the weekends with AMI were included. Weekend admissions started from Saturday, 12:01 AM, through Sunday, 11:59 PM. Urban and rural hospitals were segregated using the included NIS variable, which designates urban and rural hospitals based on core-based statistical areas (CBSAs). Patients with the diagnosis of type II non-ST-elevation myocardial infarction (NSTEMI) or AMI from secondary causes or procedural complications and patients with missing information were excluded. The patients who were transferred to other facilities were also excluded. The International Classification of Diseases (ICD)-10 Clinical Modification (CM) codes were used to identify patients hospitalized for ST-elevation myocardial infarction (STEMI) and type I NSTEMI. The ICD-10 codes used are listed in the attached Appendix.

Study variables

Variables were created using ICD-10 procedure codes to identify patients undergoing diagnostic cardiac angiograms and PCI (see Appendix). Separate variables were created for all other comorbid conditions.

Outcomes

The primary outcome was in-hospital diagnostic coronary angiography or treatment with PCI. Secondary outcomes were in-hospital mortality.

Statistical analysis

Stata Statistical Software: Release 18 (2023; StataCorp LLC, College Station, Texas, United States) was used to analyze the results. Univariable and multivariable linear regression analyses calculated means for continuous variables. Univariable logistic regression analysis computed unadjusted odds ratios (ORs) for categorical and dichotomous variables. A separate univariable logistic regression analysis identified statistically significant variables with a p-value below 0.02. Those variables were then included in the multivariate logistic regression analysis to adjust for potential confounders.

Proportions were compared using the chi-squared test and presented as numbers and percentages. Continuous variables were assessed with the Student's t-test and presented as mean with standard deviation. All p-values obtained were two-sided, with a significance threshold set at 0.05.

## Results

Out of 35 million weighted discharges included in the NIS 2019, 161,625 were admitted over the weekend with AMI. Of these patients, 47,665 (29.5%) were discharged with the primary diagnosis of STEMI and 113,960 (70.5%) with NSTEMI. The mean age of the patients included in the study was 66.5 years. Caucasian was the predominant race (114,830 (71%), African American 17,910 (11%), and Hispanic 13,920 (8.6%)), and the study included 58,690 (36%) female patients. About 93% of the patients were treated in urban and 7% in rural US community hospitals. Patients treated at the rural hospitals were more likely to be Caucasian and on Medicare. The distribution of other baseline variables was similar, as summarized in Table 1.

**Table 1:** Patient characteristics STEMI: ST-elevation myocardial infarction; NSTEMI: non-ST-elevation myocardial infarction; HMO: health maintenance organization; PCI: percutaneous coronary intervention

Patient characteristics	Urban	Rural	p-value
No. (%) of patients			
Women, no. (%)	54,105 (36)	4585 (40)	<0.001
Age, mean (SD)	66.4 (8.8)	67.5 (3.3)	<0.001
STEMI, no. (%)	44,810 (94)	2855 (5.9)	<0.05
NSTEMI, no. (%)	105,355 (92.4)	8605 (7.5)	<0.05
Race/ethnicity, no. (%)			<0.001
Caucasian	105,060 (71)	9770 (88)	
African American	17,115 (11.7)	795 (7.2)	
Hispanic	13,715 (9.4)	205 (1.8)	
Asian or Pacific Islander	4885 (3.3)	60 (0.5)	
Native American	810 (0.5)	125 (1.1)	
Other	4750 (3.2)	70 (0.6)	
Charlson Comorbidity Index score, no. (%)			<0.001
1	36,355 (24)	2640 (23)	
2	37,550 (25)	2950 (26)	
3	26,985 (17)	2155 (19)	
>4	51,050 (34)	3715 (32)	
Median annual income in patients' zip code, no. (%)			<0.001
$1-45,999	42,485 (28.5)	6520 (58)	
$46,000-58,999	39,055 (26)	3330 (29)	
$59,000-78,999	38,740 (26)	1165 (10)	
>$79,000	29,580 (19.6)	240 (2)	
Insurance type, no. (%)			<0.001
Medicare	83,165 (57)	6990 (63)	
Medicaid	14,680 (9.8)	1020 (9.2)	
Private HMO	41,255 (28)	2420 (22)	
Self-pay	7530 (5.2)	580 (5.3)	
Hospital characteristics			
Hospital region, no. (%)			<0.001
Northeast	25,400 (17)	1490 (13)	
Midwest	32,065 (21)	3480 (30)	
South	62,950 (41)	5585 (48)	
West	31,245 (20)	905 (7.9)	
Hospital bed size, no. (%)			<0.001
Small	31,205 (21)	530 (4.6)	
Medium	48,605 (32)	1075 (9.4)	
Large	71,550 (47)	9855 (86)	
Early diagnostic angiogram	79,850 (54)	5330 (46)	<0.001
Early PCI	58,290 (40)	3845 (33)	<0.001
Hospital teaching status, no. (%)	1,239,520 (80)	0	<0.001

In-hospital diagnostic angiography and PCI

A total of 123,135 (76%) AMI patients underwent diagnostic coronary angiography over the weekends, including 43,105 (90%) STEMI and 80,030 (70%) NSTEMI patients. After adjusting for patient- and hospital-level variables, patients admitted to a rural hospital were less likely to undergo diagnostic angiography than those at urban centers (adjusted OR (aOR), 0.69; CI, 0.57-0.83; p<0.001).

PCIs were performed on 84,025 (52%) AMI patients during weekends, including 39,005 (82%) STEMI and 45,020 (39%) NSTEMI patients. Like diagnostic angiography, patients in rural hospitals had lesser odds of undergoing percutaneous intervention than in urban hospitals (aOR, 0.83; CI, 0.72-0.96, p 0.012). The individual aORs of diagnostic angiogram and PCI for STEMI and NSTEMI were also statistically significant, except for the odds of PCI in STEMI patients, as summarized in Table 2.

**Table 2:** Unadjusted and adjusted ORs for diagnostic angiography and PCI in rural hospitals AMI: acute myocardial infarction; STEMI: ST-elevation myocardial infarction; NSTEMI: non-ST-elevation myocardial infarction; PCI: percutaneous coronary intervention; OR: odds ratio

	AMI		STEMI		NSTEMI	
	Unadjusted OR (95% CI)	Adjusted OR (95% CI)	Unadjusted OR (95% CI)	Adjusted OR (95% CI)	Unadjusted OR (95% CI)	Adjusted OR (95% CI)
Diagnostic coronary angiogram	0.61 (0.53-0.72)	0.69 (0.57-0.83)	0.79 (0.56-1.13)	0.48 (0.29-0.77)	0.62 (0.53-0.72)	0.72 (0.60-0.87)
PCI	0.75 (0.66-0.85)	0.83 (0.72-0.96)	1.09 (0.84-1.41)	0.90 (0.66-1.24)	0.73 (0.64-0.84)	0.82 (0.70-0.96)

In-hospital mortality

A total of 7545 (4.6%) patients with AMI died during weekends, 3695 (7.7%) with STEMI and 3850 (3.4%) with NSTEMI, as summarized in Table 3. The unadjusted and adjusted ORs for the difference in AMI mortality over the weekends between urban and rural hospitals were not statistically significant (aOR, 1.08; CI, 0.85-1.4; p 0.52). Similar results were obtained for STEMI and NSTEMI when adjusted for patient- and hospital-level variables, as outlined in Table 4.

**Table 3:** Mortality numbers in urban and rural hospitals over the weekend AMI: acute myocardial infarction; STEMI: ST-elevation myocardial infarction; NSTEMI: non-ST-elevation myocardial infarction

	Total	Urban	Rural
Total AMI no. (%)	7545/161,625 (4.6%)	7075/150,165 (4.7%)	470/11,460 (4.1%)
STEMI no. (%)	3695/47,665 (7.7%)	3495/44,810 (7.8%)	200/2855 (7%)
NSTEMI no. (%)	3850/113,960 (3.4%)	3580/105,355 (3.4%)	270/8605 (3.1%)

**Table 4:** Adjusted and unadjusted ORs of mortality in patients with AMI, STEMI, and NSTEMI in rural hospitals AMI: acute myocardial infarction; STEMI: ST-elevation myocardial infarction; NSTEMI: non-ST-elevation myocardial infarction; OR: odds ratio

Mortality	AMI		STEMI		NSTEMI	
	Unadjusted OR (95% CI)	Adjusted OR (95% CI)	Unadjusted OR (95% CI)	Adjusted OR (95% CI)	Unadjusted OR (95% CI)	Adjusted OR (95% CI)
	0.86 (0.70-1.06)	1.08 (0.85-1.38)	0.89 (0.64-1.23)	0.91 (0.64-1.28)	0.92 (0.70-1.21)	0.88 (0.67-1.17)

Early diagnostic angiogram and PCI

Early procedures were defined as the ones performed within the first 24 hours of hospital admissions. Patients treated for AMI in rural hospitals had lesser odds of undergoing early diagnostic coronary angiography (aOR, 0.79; CI, 0.67-0.95; p<0.05) and percutaneous coronary angiogram (aOR, 0.78; CI, 0.66-0.92; p<0.05) compared to the urban hospital.

On separate analyses for STEMI and NSTEMI, the results for patients with NSTEMI undergoing early diagnostic angiogram and patients with STEMI undergoing early PCI were not statistically significant, as seen in Table 5.

**Table 5:** Unadjusted and adjusted ORs of early diagnostic angiogram and PCI in rural hospitals AMI: acute myocardial infarction; STEMI: ST-elevation myocardial infarction; NSTEMI: non-ST-elevation myocardial infarction; PCI: percutaneous coronary intervention; OR: odds ratio

	AMI		STEMI		NSTEMI	
	Unadjusted OR (95% CI)	Adjusted OR (95% CI)	Unadjusted OR (95% CI)	Adjusted OR (95% CI)	Unadjusted OR (95% CI)	Adjusted OR (95% CI)
Early diagnostic angiogram	0.75 (0.65-0.87)	0.79 (0.67-0.95)	0.64 (0.45-0.90)	0.48 (0.31-0.73)	0.83 (0.72-0.97)	0.92 (0.77-1.09)
Early PCI	0.76 (0.67-0.87)	0.78 (0.67-0.92)	0.83 (0.62-1.09)	0.79 (0.58-1.08)	0.80 (0.69-0.94)	0.81 (0.68-0.95)

## Discussion

The disparities in AMI mortality between urban and rural hospitals have been studied before. Various studies indicate a consistent pattern of increased mortality and reduced utilization of cardiac catheterization, thrombolysis, and PCI in rural hospitals [16-18]. Additionally, previous research has also shown increased AMI mortality on the weekends, irrespective of the hospital location [19,20]. This observational study evaluated the utilization of diagnostic coronary angiography and PCIs in rural hospitals during the weekends. The findings revealed that AMI patients treated in rural hospitals are less likely to undergo these procedures. When these procedures are performed, the odds of conducting them within the first 24 hours are lower in rural settings. Notably, this trend did not affect weekend mortality of AMI in rural areas, as the results were not statistically significant.

Current guidelines recommend early reperfusion therapy for STEMI with a D2B time of less than 90 minutes and an invasive coronary angiogram within 24 hours of NSTEMI diagnosis [21]. However, achieving these goals is more challenging in rural hospitals with limited resources and access to PCI-capable facilities. STEMI patients are directly transferred to the closest PCI-capable hospitals by emergency medical services, and patients diagnosed with new in-house STEMI in rural hospitals are also emergently transferred to the nearest PCI-capable facility. These transfers often cause unnecessary delays. This effect is further exponentiated on the weekends when access to catheterization labs and interventional cardiologists is further limited. Previous research validated this study in discovering lesser odds of undergoing a diagnostic angiogram on the weekend in the United States [22]. This presenting study also showed lesser odds of having these procedures in rural hospitals. When these procedures were performed, patients in rural hospitals had significantly lesser odds of undergoing diagnostic angiograms and percutaneous interventions within the recommended timeframe.

Interestingly, some earlier studies reported no difference in AMI mortality on weekends despite fewer invasive procedures and treatments [23-25], while others showed increased deaths [19,20,26,27]. Meta-analysis of studies over two decades indicated a trend of rising mortality in rural America [28]. Despite having a higher mortality rate in rural hospitals, this presenting study showed no statistically significant difference in mortality rates between urban and rural hospitals over the weekends.

The incidence of future cardiovascular events tends to be higher in rural areas due to the continuous urban migration of young people and rural migration of retirees [29]. Access to a subspecialist like a cardiologist is vital, as outcomes are better in counties with higher cardiologist density [16]. With over 60% of cardiologists aged 55 and above [30], coupled with a growing patient population attributed to extended life expectancy from novel medications and procedures, rural hospitals face a shortage of cardiologists [31], resulting in higher mortality rates in recent surveys [32]. This trend will directly affect weekend mortality of acute coronary syndrome [33].

Limitations

The study was performed on a retrospective database where inclusion criteria depended on providers' reliable coding for accurate ICD codes. Patients with missing information were excluded, which may infer a selection bias. The database included all-cause mortality and not cardiovascular-specific mortality.

## Conclusions

This study showed a trend toward decreased utilization of diagnostic angiograms and PCI in rural hospitals on weekends. The odds of performing these procedures within the recommended timeframe were also less. Despite these disparities, there was no statistically significant difference in AMI mortality over the weekend between rural and urban centers. We suggest prioritizing resource allocation in rural community hospitals. Implementing new policies is essential to encourage clinical personnel to relocate and enhance healthcare coverage in rural communities.

## Appendices

**Table 6:** ICD-10 diagnostic and procedure codes ICD: International Classification of Diseases; STEMI: ST-elevation myocardial infarction; NSTEMI: non-ST-elevation myocardial infarction; CAD: coronary artery disease; CHF: congestive heart failure; MI: myocardial infarction; PCI: percutaneous coronary intervention; PAD: peripheral vascular disease

Diagnosis/procedure	ICD-10 Clinical Modification codes
STEMI	I2101, I2102, I2109, I2111, I2119, I2121, I2129, I2130
NSTEMI	I214
Diabetes mellitus	E1010, E1011, E10618, E10620, E10621, E10622, E10628, E10630, E10638, E10641, E10649, E1065, E1069, E108, E109, E1100, E1101, E11618, E11620, E11621, E11622, E11628, E11630, E11638, E11641, E11649, E1165, E1169, E118, E119, E1300, E1301, E13618, E13620, E13621, E13622, E13628, E13630, E13638, E13641, E13649, E1365, E1369, E138, E139, E1310, E1311
Hypertension	I10, I110, I119, I129, I120, I130, I1310, I1311, I132
Hyperlipidemia	E782, E784, E785
Obesity	E6601, E6609, E661, E662, E668, E669
Smoking	Z87891, F17200, F17201, F17203, F17208, F17209, F17211, F17213, F17220, F17221, F17223, F17228, F17229, F17290, F17291, F17293, F17298, F17299, Z720, F17210, F17218, F17219, Z720, F17210, F17218, F17219
CAD	I2510, I25110, I25111, I25118, I25119
Systolic heart failure	I5020, I5021, I5022, I5023
Diastolic heart failure	I5030, I5031, I5032, I5033
Combine CHF	I5040, I5041, I5042, I5043
History of MI	I252
History of PCI	Z955, Z9861
PAD	I739
Diagnostic angiogram	B210010, B2100ZZ, B210110, B2101ZZ, B210Y10, B210YZZ, B211010, B2110ZZ, B211110, B2111ZZ, B211Y10, B211YZZ, B2150ZZ, B2151ZZ, B215YZZ, B2160ZZ, B2161ZZ, B216YZZ, B2050ZZ, B2051ZZ, B205YZZ, B2060ZZ, B2061ZZ, B206YZZ, B2000ZZ, B2001ZZ, B200YZZ, B2010ZZ, B2011ZZ, B201YZZ, 4A023N7, 4A023N8
PCI	0210344, 02103D4, 0211344, 02113D4, 0212344, 02123D4, 0270346, 027034Z, 0270356, 027035Z, 0270366, 027036Z, 0270376, 027037Z, 02703DZ, 02703E6, 02703EZ, 02703F6, 02703FZ, 02703G6, 02703GZ, 02703T6, 02703TZ, 02703Z6, 02703ZZ, 0271346, 027134Z, 0271356, 027135Z, 0271366, 027136Z, 0271376, 027137Z, 02713DZ, 02713E6, 02713EZ, 02713F6, 02713FZ, 02713G6, 02713GZ, 02713T6, 02713TZ, 02713Z6, 02713ZZ, 02713D6, 0272346, 027234Z, 027235Z, 0272366, 027236Z, 0272376, 027237Z, 02723DZ, 02723E6, 02723EZ, 02723F6, 02723FZ, 02723G6, 02723GZ, 02723T6, 02723TZ, 02723Z6, 02723ZZ, 0272356, 02723D6, 0273346, 027334Z, 027335Z, 0273366, 027336Z, 0273376, 027337Z, 02733DZ, 02733E6, 02733EZ, 02733F6, 02733FZ, 02733G6, 02733GZ, 02733T6, 02733TZ, 02733Z6, 02733ZZ, 0273356, 02733D6, 02C03Z6, 02C03ZZ, 02C13Z6, 02C13ZZ, 02C23Z6, 02C23ZZ, 02C33Z6, 02C33ZZ, 02H03DZ, 02H03YZ, 02H13DZ, 02H13YZ, 02H23DZ, 02H23YZ, 02H33DZ, 02H33YZ, 02Q03ZZ, 02Q13ZZ, 02Q23ZZ, 02Q33ZZ, 02U03JZ, 02U13JZ, 02U23JZ, 02U33JZ
